# An audit of mother to child HIV transmission rates and neonatal outcomes at a tertiary hospital in South Africa

**DOI:** 10.1186/s13104-019-4617-1

**Published:** 2019-09-18

**Authors:** Ghad Benali, Tanusha Ramdin, Daynia Ballot

**Affiliations:** Division of Neonatology, Department of Paediatrics and Child Health, Charlotte Maxeke Johannesburg Academic Hospital, University of the Witwatersrand, Johannesburg, South Africa

**Keywords:** HIV positive mother, HIV exposed neonate, HIV prophylaxis, ANC visit, HIV transmission

## Abstract

**Objective:**

The aim of this study was to explore the prevalence of congenital HIV infection of neonates at Charlotte Maxeke Johannesburg Academic Hospital (CMJAH) between 2015 and 2017, as well as compare the HIV PCR positive and HIV PCR negative neonates.

**Results:**

A total number of 1443 HIV exposed neonates was examined for the study period out of a total of 5029 admissions (HIV exposure 28.6%) The study found that the rate of HIV transmission at birth was 2.52%. The majority of infants had low birth weight and were also born prematurely. These results show that, despite the introduction of the extended mother to child transmission programme, HIV transmission is high.

## Introduction

Human Immunodeficiency Virus (HIV) disease has been, and continues to be, a health challenge to overcome globally. One of the main challenges with HIV is the high prevalence of HIV infections in mothers and children. Nearly half of all HIV-infected adults are women of child bearing age, and as a result of this, the majority of HIV infections in children are a result of mother to child transmission (MTCT) [1]. According to the Foundation for AIDS Research, 91% of the world’s HIV-positive children live in sub-Saharan Africa [1]. The majority of MTCT of HIV takes place during pregnancy and/or delivery; therefore, interventions to prevent mother-to-child transmission are urgently needed to reduce the future incidence of paediatric HIV [2].

The HIV transmission rate has been shown to be twice as high in Africa as in Europe. This difference is not yet fully understood, but contributory factors include: primary HIV infection occurring during pregnancy [3]; lower maternal CD4 count; differences in use of antiretroviral (ARV) drugs (e.g. less extensive availability, lower adherence rates, higher drug abuse rates); differences in virulence of the virus according to geographical origin; co-existence with other sexually transmitted diseases; concomitant infections in the mother; invasive intra-partum procedures (e.g. fetal scalp electrodes and forceps); chorioamnionitis [3]; vaginal delivery; rupture of membranes (especially if delivery is more than 4 h after the membranes ruptured); advanced maternal age; lack of prevention of mother-to-child transmission (PMTCT) services; and mixed feeding regimes [4, 5]. This shows that the transmission of HIV from mother to child is a multi-factorial event. A number of these factors are unable to be controlled or reduced in a meaningful manner in a resource limited setting, but the availability of ARVs and preventative MTCT services are two areas that can be influenced.

The transmission of HIV can be trans-placental during early or late gestation, and the prevalence of trans-placental transmission is high, especially in African countries as compared with global rates [5, 6]. A number of researchers have shown a higher mortality rate among babies born to HIV positive mothers, as compared to those born to seronegative mothers [7]. Prematurity, low birth weight, and intrauterine growth retardation, are also higher in HIV exposed infants [7]. HIV polymerase chain reaction (PCR) screening was introduced in South Africa in 2015 as part of the extended mother to child transmission (EMTCT) programme. The aim of this study was to review HIV exposed neonates admitted to a tertiary hospital in Johannesburg, South Africa, after the introduction of EMTCT.

## Main text

This was a retrospective, cross sectional descriptive study conducted in the neonatal unit at Charlotte Maxeke Johannesburg Academic Hospital (CMJAH) from 1st of January 2015 to the 31st of December 2017. The study population included all neonates admitted to the neonatal unit during the study period. Mothers were routinely tested for HIV infection during antenatal care and at the time of delivery. All HIV exposed neonates were tested at birth using an HIV PCR. This was a non-random sample. All HIV exposed neonates were eligible for inclusion. The final sample comprised those neonates with a known birth HIV PCR result who were admitted to the neonatal unit. We did not report on healthy HIV exposed neonates who were not admitted. The study site was a tertiary referral hospital with a large number of high risk obstetric cases, uncomplicated pregnancies were managed at midwife obstetric units.

This was a secondary analysis of an existing database. The data of all patients was collected by attending medical staff on discharge using a standard data collection form for the purpose of quality control. Data was managed in a database using the Research Electronic Data Capture (REDCap) [8]. We only collected information on maternal HIV infection and provision of HIV prophylaxis to the neonate (coded Yes/No/Unknown), as well as the result of the neonatal birth PCR. Additional important maternal information such as CD4 count, viral load, ARV therapy, compliance and AIDS related illness was not available for analysis from the database.

Neonates were divided on the basis of the PCR test into birth HIV positive and negative. Outcome, clinical and demographic data was compared between the two groups.

Data was analysed using IBM SPSS 25. Missing values were excluded in the analysis of each variable. Continuous variables, such as birth weight and gestational age, were described using mean and standard deviation as data was normally distributed. On the other hand, categorical variables, such as gender and mode of delivery, were described using percentages.

Categorical variables were compared using Chi Square. Continuous variables were compared using an independent sample t test, as the data was normally distributed. A p value ≤ 0.05 was considered statistically significant.

**Fig. 1:**
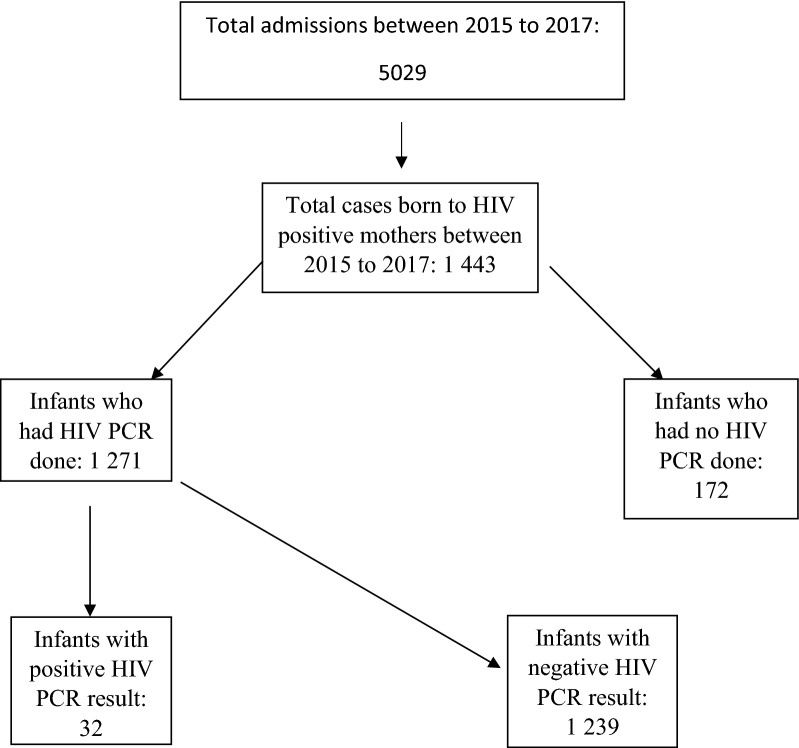
Breakdown of study sample

There were 5029 neonatal admissions during the study period, of which 1 443 (28.6%) were HIV exposed. The results were broken down as shown in Fig. 1.

From the 1 443 cases, only 1 271 cases had a birth PCR done (88.1%). Overall, HIV transmission at birth was 32/1271 (2.52%).

There were significantly more neonates with congenital HIV born to mothers who had syphilis (p = 0.006). There were no major differences in mortality (p = 0.408), and there were also no differences in other aspects such as ANC, maternal chronic illnesses, mode of delivery, major birth defect and neonatal sepsis (see Table 1). The study also revealed that there were no differences in gestational age, birth weight, length of stay in hospital and the maternal age between the two groups under study (see Table 2).

**Table 1:** Maternal and neonatal characteristics compared between HIV PCR positive and negative neonates

Characteristic	HIV PCR negative (1239 neonates)	HIV PCR positive (32 neonates)	p value
Male	666/1234 (54.0%)	14/32 (43.8%)	0.252
Any antenatal care	957/1136 (84.2%)	21/28 (75.0%)	0.187
Normal vaginal delivery	570/1176 (48.5%)	18/29 (62.1%)	0.348
Multiple gestation	162/1227 (13.2%)	3/30 (10.0%)	0.608
Nulliparous mother	148/1065 (13.9%)	3/27 (11.1%)	0/944
Chorioamnionitis	318/1085 (3.5%)	1/27 (3.7%)	0.955
Maternal hypertension	143/1091 (13.1%)	3/27 (11.1%)	0.761
Maternal tuberculosis	28/1113 (2.5%)	1/28 (3.6%)	0.726
Maternal diabetes mellitus	12/1139 (1.1%)	0/32 (0%)	0.578
Maternal syphilis	44/1135 (3.9%)	4/28 (14.3%)	0.006
Bag mask ventilation at birth	307/1239 (24.7%)	9/30 (30.0%)	0.523
Ventilatory support			
CPAP	410/1037 (39.5%)	11/24 (45.8%)	0.533
Mechanical ventilation	207/1039 (19.9%)	3/24 (12.5%)	0.367
Life threatening congenital abnormality	77/1224 (6.3%)	1/32 (3.1%)	0.464
Culture proven sepsis on or before day 3	55/1211 (4.5%)	1/30 (5.3%)	0.753
Culture proven sepsis after day 3	227/1227 (18.5%)	9/32 (28.1%)	0.168
Neonatal jaundice requiring phototherapy	364/1196 (30.4%)	8/31 (25.8%)	0.586
Necrotising enterocolitis (stage 2 or 3)	66/1220 (5.4%)	1/30 (3.3%)	0.618
Hypoxic ischemic encephalopathy	79/822 (9.6%)	1/23 (4.3%)	0.395
Died	136/1239 (11.0%)	5/32 (15.6%)	0.408

**Table 2:** Characteristics of 1271 neonates born to HIV positive mothers

Characteristic	HIV PCR negative	HIV PCR positive	p value
Gestational age	34.7 weeks (SD 4.1)	32.6 weeks (SD 3.9)	0.786
Birth weight (kg)	2.04 kg (SD 0.9)	1.96 kg (SD 0.78)	0.615
Length of stay	17.7 days (SD 21.6)	18.7 days (SD 19.9)	0.783
Maternal age (years)	30.4 years. (SD 5.9)	30.5 years. (SD 6.8)	0.128

This review of HIV exposed neonates, admitted to a tertiary neonatal unit, after the introduction of EMTCT found that 28.6% were HIV exposed, while 2.52% of neonates had congenital HIV. In 2014, the National Health Laboratory Service reported that 1.7% of HIV exposed infants tested between 4 and 8 weeks postnatal age were HIV PCR positive [9]. This shows that despite the programs to prevent MTCT the rate of transmission remains quite high. However, it should be kept in mind that the study focused only on sick neonates who required admission to the hospital. Future studies should evaluate the reasons for failure of these MTCT interventions. There were no significant differences between HIV PCR positive and HIV PCR negative babies, especially when compared with the high-risk babies and the outcome, which was unexpected.

About three quarters of the mothers attended ANC. Unexpectedly, this attendance was almost the same between the mothers of the HIV PCR positive (75%) and HIV PCR negative (84.2%) babies. However, it is not clear whether those mothers also started visiting the ANC before or after 28 weeks of gestation. This number shows that there is still a large number of mothers who are not aware of the importance of attending the ANC, despite the efforts made by the government and other organizations to educate women.

A large number of mothers had a variety of pre-existing diseases, including hypertension, diabetes mellitus, tuberculosis, syphilis and chorioamnionitis. Unpredictably, there was no increase in the rate of transmission of HIV among those neonates born to the mothers who had these diseases, except in the case of syphilis. The study shows high congenital HIV infection among neonates born to mothers who were infected with HIV and syphilis; which is to be expected as syphilis is a co-morbidity with HIV [10].

In addition, there were no differences in neonatal sepsis, in both cases of early and late sepsis. Early sepsis was shown to be 4.5% in HIV PCR negative neonates, compared to 5.3% in HIV PCR negative neonates (p = 753). Late sepsis was 18.5% in HIV PCR negative, while in HIV PCR positive neonates, it was shown to be 28.1% (p = 0.168). These high percentages show that both groups are vulnerable to a variety of neonatal infections due to maternal HIV disease [11]. In every instance, a mother passes on her immunity to her baby. However, when the mother’s immunity is already low, as in the case of an HIV positive mother, especially with a concurrent disease; the baby’s immunity level also becomes compromised, more so because the infant also has HIV disease; hence resulting in increased vulnerability to infection [11].

The majority of the babies under study were born with low weight. This is mainly as a result of the gestational age, as most of the infants were born prematurely. This high number of premature births may be attributed to the HIV status of the mothers. Because HIV is a chronic illness, it contributes to an increased metabolic demand. This may be a contributing factor to a decreased supply of intra-uterine nutrition to the baby; thereby leading to low birth weight [12]. In addition, there was no significant difference in the continuous variables between neonates who were HIV PCR positive at birth, as compared to those who were negative (see Table 2). Of all the neonates under study, 26.6% died, with 15.6% being HIV PCR positive neonates.

## Limitations

This analysis was limited to data collected in an existing database and only considered sick neonates who were admitted to the neonatal unit. We did not determine the HIV transmission rate in the whole neonatal population. Neither did we evaluate reasons for failure of the EMTCT program.

## Data Availability

The data will be made available upon reasonable request to the corresponding author

## Abbreviations

AIDS: acquired immunodeficiency syndrome
ANC: antenatal care
ARV: antiretroviral
CD4: cluster of differentiation 4
CMJAH: Charlotte Maxeke Johannesburg Academic Hospital
CPAP: Continuous Positive Airways Pressure
EMTCT: extended mother to child transmission
MTCT: mother to child transmission
PCR: polymerase chain reaction
PMTCT: prevention of mother to child transmission
REDCap: Research Electronic Data Capture
